# Two Vascular Beds, One Mystery: Concurrent Renal Vein Thrombosis and Ischemic Stroke

**DOI:** 10.51894/001c.164569

**Published:** 2026-07-31

**Authors:** Alex Azzouz, Umer Javaid, Preeti Misra

**Affiliations:** 1 Trinity Health - Livonia INTRODUCTION

## 62

### Introduction

Concurrent arterial and venous thromboses are uncommon and should prompt evaluation for underlying hypercoagulable disorders. While antiphospholipid syndrome, malignancy, and inherited thrombophilias are well-recognized causes, some patients present with thrombotic events in unusual locations without a definitive diagnosis. We present a case of simultaneous renal vein thrombosis and ischemic stroke in a patient with unexplained hypercoagulable state.

### Case Description

A 58 year-old female with history of hypertension, diabetes mellitus, and prior stroke presented with altered mental status and weakness. Initial evaluation revealed DKA with evidence of pneumonia and bilateral pyelonephritis. CT abdomen/pelvis incidentally demonstrated a right renal vein thrombosis extending into the IVC. During the same hospitalization, brain MRI revealed multiple ischemic infarcts within the right middle cerebral artery territory, representing a concurrent arterial thrombotic event. Given the atypical location of venous thrombosis and presence of both arterial and venous events, a hypercoagulable evaluation was pursued. Laboratory testing demonstrated reduced protein S levels, elevated anticardiolipin IgG antibodies, and a positive lupus anticoagulant. However, these findings were obtained in the setting of acute thrombosis and anticoagulation, raising concern for possible false-positive results. Repeat testing by hematology later in the clinical course was reassuring and did not confirm a definitive hypercoagulable disorder. Evaluation for underlying malignancy did not identify a clear neoplastic source of thrombosis.

### Discussion

The coexistence of arterial and venous thromboses raises concern for systemic hypercoagulable disorders. Renal vein thrombosis extending into the IVC is an uncommon thrombotic presentation and further increased suspicion for an underlying systemic process. Hematologic evaluation therefore focused on conditions known to produce both arterial and venous thrombotic events, particularly antiphospholipid syndrome, inherited thrombophilias, and malignancy-associated hypercoagulability. Evaluation included testing for inherited thrombophilia such as Factor V Leiden mutation, which was negative. Because laboratory abnormalities detected during acute thrombosis or anticoagulation may be transient, repeat testing was performed to determine whether these findings represented persistent thrombophilia. Despite comprehensive hematologic evaluation, repeat testing did not confirm a definitive hypercoagulable diagnosis. This case highlights the diagnostic complexity of coexisting arterial and venous thromboses and demonstrates that a clear etiology may remain uncertain even after extensive investigation.

